# Evaluation of physiological risk factors, oxidant-antioxidant imbalance, proteolytic and genetic variations of matrix metalloproteinase-9 in patients with pressure ulcer

**DOI:** 10.1038/srep29371

**Published:** 2016-07-11

**Authors:** Khlifi Latifa, Sahli Sondess, Graiet Hajer, Ben-Hadj-Mohamed Manel, Khelil Souhir, Bouzidi Nadia, Jaballah Abir, Ferchichi Salima, Miled Abdelhedi

**Affiliations:** 1 Biochemistry Laboratory CHU Farhat HACHED, Sousse, Tunisia

## Abstract

Pressure ulcer (PU) remains a common worldwide problem in all health care settings, it is synonymous with suffering. PU is a complex disease that is dependent on a number of interrelated factors. It involves multiple mechanisms such as physiological risk factors, chronic inflammation, oxidant–antioxidant imbalance and proteolytic attack on extracellular matrix by matrix metalloproteinases (MMP). Therefore, we propose that these wounds lead to molecular variations that can be detected by assessing biomarkers. In this study, we aimed to evaluate the major clinical elements and biological scars in Tunisian patients suffering from PU. Consistently, non-healing wound remains a challenging clinical problem. The complex challenges of the wound environment, involving nutrient deficiencies, bacterial infection, as well as the critical role played by inflammatory cells, should be considered because of their negative impact on wound healing. In addition, an imbalance between pro-oxidants and antioxidant systems seems to be more aggravated in patients with PU compared to healthy subjects. Of interest, this study provides further evidence to support a core role of the biological activity of MMP-9 in the pathogenesis of PU and indicates that the MMP9-1562 C/T (rs 3918242) functional polymorphism is associated with protection against this disease.

Being bedridden can be very difficult for many patients to adjust to, it can also cause other health problems^1^. The most common health risk for bedridden patients is pressure ulcer (PU)^2^. PU is commonly termed as bedsore, reflects a localized damage to the skin and underlying tissues, as a result of prolonged pressure higher, occurring when soft tissue is compressed between a bony prominence and an external surface. Heightened pressure, and/or in combination with torsion forces, can leads to decreased oxygen and nutrients reaching the tissues in combination with interruption of blood flow to a given area^3^,^4^.

The danger to being affected by PU is well known and it is a long standing problem. PU causes pain, increase morbidity and mortality rates, a high financial burden for the healthcare system and diminishes patient’s quality of life. It can develop quickly and is often difficult to treat^5^. PU is synonymous with suffering.

Despite diverse research, accumulated knowledge concerning these wounds and developing treatments, the number of PU is still increasing^6^,^7^,^8^,^9^. The National Pressure Ulcer Advisory Panel says the incidence ranges from 0.4% to 38% in hospitals, from 2.2% to 23.9% in skilled nursing facilities and from 0% to 17% for home health agencies^10^. There are wide variations between intra- and inter-country from 3.9% to over 18%^11^,^12^. However, the optimal incidence rate may vary depending on patient case mix, severity of illness, methodology for each survey and other contextual factors^13^.

The formation and molecular mechanisms underlying causes of PU are quite complex, with multiple influencing factors^14^. Extensive studies have identified several factors that increase the risk of PU^10^. The list of factors associated with PU risk is long, numbering more than 100, and includes previous medical events, patient demographic characteristics, anthropometric characteristics, functional status, physiological status, nutritional status, infectious agents, cognition, psychological status, immunological factors, and more^3^,^15^.

Usually, any chronic wound damages the tissue and affects the local environment. The host’s response to wounding involves a series of coordinated interactions between diverse immunological and biological systems. This process can lead to a cascade of carefully and precisely regulated steps and events that correlate with the appearance of both resident and migratory cell populations, as well as the extracellular matrix and the action of soluble mediators such as growth factors and cytokines^16^.

Additionally, a variety of studies that analyzed blood, fluids and biopsies collected from chronic, nonhealing wounds found elevated levels of inflammatory cytokines, elevated levels of matrix metalloproteinases (MMP) and low levels of growth factors compared with acute, healing wounds. These signaling pathways might impede the wound healing process^17^,^18^. Moreover, prolonged, elevated levels of the gelatinase subset, MMP-9, a member of the MMP family of zinc-endopeptidases, are found in patients with chronic wounds that appear to have detrimental effects on wound healing^19^,^20^.

In particular, MMP-9 expression and/or activity under non-remodeling conditions are low, but are highly inducible following injury in response to a variety of damaging agents or myopathic conditions^18^. It seems likely that MMP-9 activity plays several key roles in extracellular matrix remodeling and immune cell migration during wound repair^21^. However, the protein is secreted as a pro-enzyme, activated when the N-terminus pro-domain unfolds and is cleaved by activator proteases exposing the catalytic domain, adding a significant post-transcriptional element to its adjustment^22^. Transcriptional regulation of MMP-9 by various inflammatory factors and developmental queues is considered to be the predominant mechanism of controlling expression of this pleiotropic molecule^21^.

Polymorphisms in the promoter of MMP-9 have been implicated in the regulation of gene expression and susceptibility to various diseases^23^. In the main, some polymorphisms have influence on gene expression, protein stability, protein activity, protein-protein interactions and some of them are associated with an increased risk for diseases. Notably, a single nucleotide polymorphism (SNP) -1562C/T MMP9 results in a loss of binding of a nuclear protein and an increase in transcriptional activity in macrophages^24^. However, little is known about association of SNP -1562C/T MMP9 with PU.

Therefore, we conducted a case control study to determine some physiological risk factors related to PU and the involvement of inflammation and oxidative stress in the development of PU. Besides, we aimed to investigate whether the MMP-9 proteolytic activity and the SNP -1562C>T in the promoter region of MMP9 gene are associated with the risk of developing PU in bedridden Tunisian patients.

## Results

### Clinical and epidemiological characteristics of the subjects suffering from pressure ulcer

Demographic and pressure injury risk assessment details of enrolled subjects suffering from PU are summarized in Supplementary Table S1. From those subjects, 50% were admitted to the emergency unit (27% in coma). The patients reported having at least 1 chronic health condition in addition to PU and this indicates polypathology which aggravate their state and sphincter incontinence. Indeed, there are several factors that increase the risk of developing PU in our patients such as impaired mobility, health conditions, poor nutrition, sepsis, urinary and fecal incontinence, lack of healthcare and consciousness.

**Figure 1:**
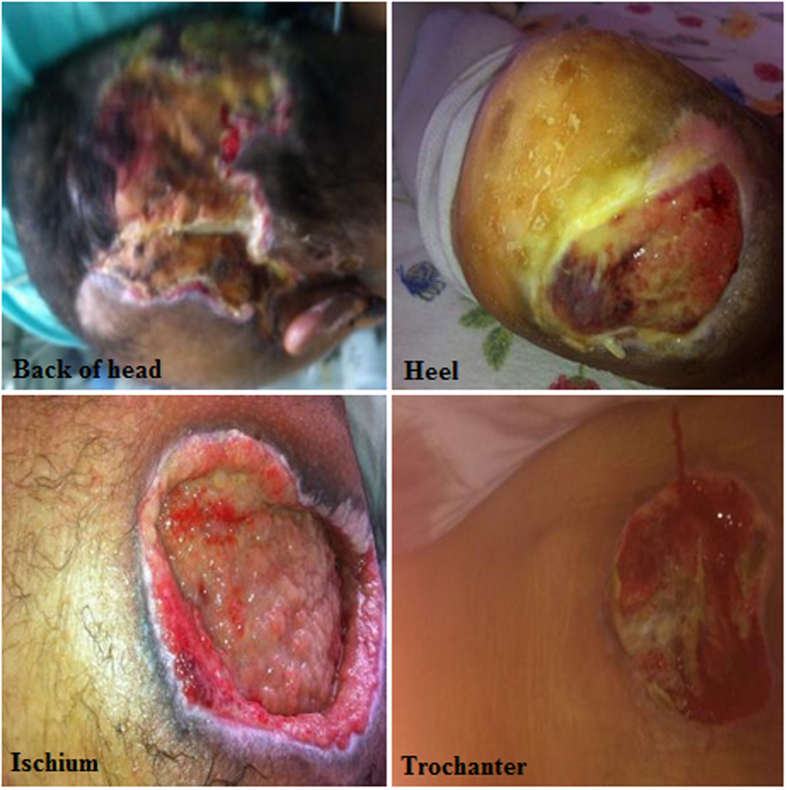
Common pressure ulcer locations.

**Figure 2:**
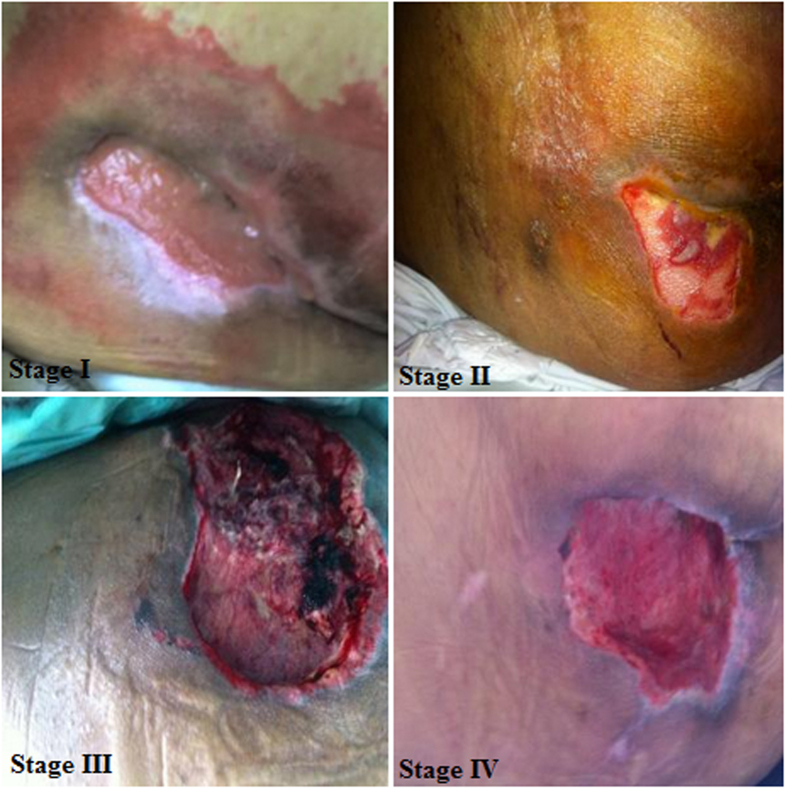
Stages of pressure ulcer.

**Figure 3:**
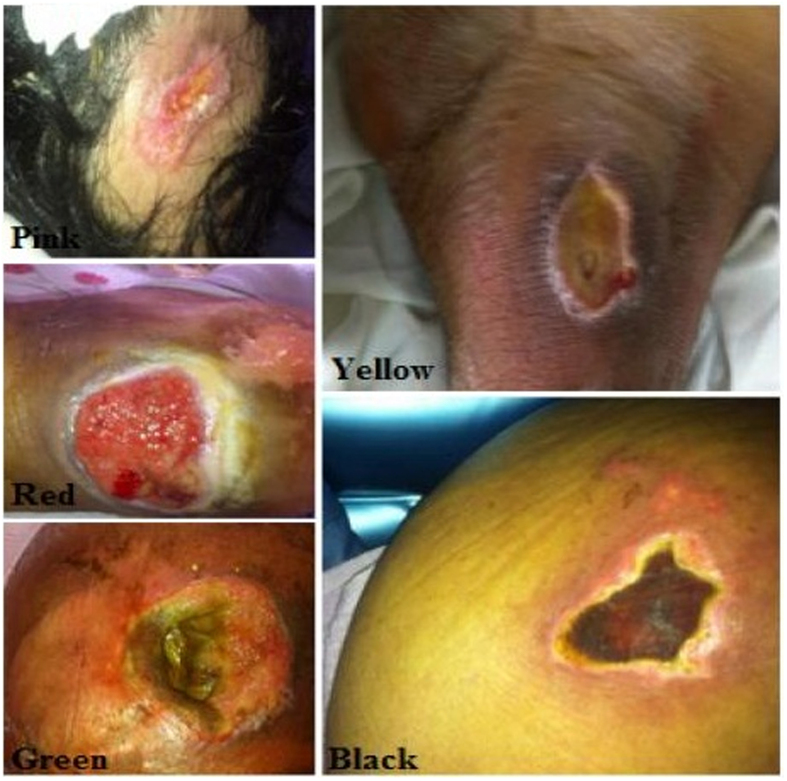
Colorielle classification scale wounds.

PU can develop in any area of the body and the damage usually occurs over bony prominences. Our patients have generated 25% to 70% of wounds in the pelvic girdle (lower back, buttocks and greater trochanter), 5% to 55% of wounds occur in the lower legs, heel and lateral area of the foot and 5% to 15% occur in the upper (back of head, the headset and shoulder) (Fig. 1; Supplementary Fig. S1). PU stages (I to IV) (Fig. 2) and change color of tissue injury (Pink-Red-Green-Yellow-Black) are shown (Fig. 3).

Malnutrition may decrease the body’s ability to fight infections and have a negative impact on PU healing. The evaluation of nutritional status, using a variety of indexes and cutoff values used for anthropometric and biological assessment, demonstrates a bad nutrition state for our patients suffering from PU (Table 1).

Most patients (80%) had bacterial infections, caused mainly by gram-negative bacilli. However, the results of smear performed on surface wounds indicate that 1 to 2 species are present at the same time. During our study, it was found that enterobacteria represents the majority of isolated germ most of them are proteus mirabilis followed by stuartii providencia, pseudomonas aeruginosa, staphylococcus aureus, escherichia coli, klebsiella pneumonia, aciretobacterium baumanni and we also note the presence of staphylococcus haemolyticus, coagulase-negative staphylococci, serratia marcescens and polymorphic flora.

During stages of PU, some germs are most frequently found and the microflora of chronic wound fluctuates over time. By bacteriological examination guide carried out in the file of some patients, taking the example of results on a young sacred wound predominant gram-positive cocci (staphylococcus aureus). After week, it is the gram-negative bacilli which colonized the wound (escherichia coli and klebsiella pneumoniae). After a few weeks the two genera colonizing (staphylococcus aureus and pseudomonas aeruginosa) change after a few weeks to acetobacterium baumannii. Following our bacterial examination of a sacred necrotic wound and exposure of bone (stage IV), it is the gram-negative bacilli enterobacteriaceae colonizing the wound (klebsiella pneumoniae and pseudomonas aeruginosa). In total and according to records, 8 patients suffering from PU have presented this fluctuation. There is a limitation on this part because some patients do not present the bacteriological examination guide in their records.

The bacteria are subject to identification tests and in some cases of antibiotic sensitivity (antibiogram). The interpretation of the results of an antibiogram is extremely complex and is based on a comparison with known resistance phenotypes. For the majority of gram-negative bacilli belonging to enterobacteriaceae, which constitute the majority of germs identified in our study, amoxicillin and clavulanic acid association, the aminoglycosides, quinolones and cephalosporins may still be first-line prescribed in first intention for which there is a rise of resistance. For the gram-positive cocci represented in our study by the only staphylococcus, penicillin G, oxacilin M, amikacin (aminoglycoside) and quinolones can still be used as first intention with a wide margin of efficiency.

### Demographic and biological characteristics of study population

The epidemiological and important biochemical characteristics of the participants are presented in Table 2 and are expressed as means ± standard deviations (SD) for quantitative variables.

Briefly, differences for fasting glucose, urea, creatinine levels, protide levels, cholesterol, triglycerides, high density lipoprotein cholesterol (HDL-C), low density lipoprotein cholesterol (LDL-C), C-reactive protein (CRP), α1‐acid glycoprotein, albumin and prealbumin levels were detected between the two groups.

Subsequently, an imbalance between pro-oxidant and antioxidant systems seems to be more aggravated in patients with PU compared than healthy subjects. Serum homocysteine levels tended to be increased in patients whereas serum total antioxidant capacity (TAS) concentrations and thiobarbituric acid reactive substances (TBARS) were found lower than in controls.

### Zymographic activity of matrix metalloproteinase-9

**Figure 4:**
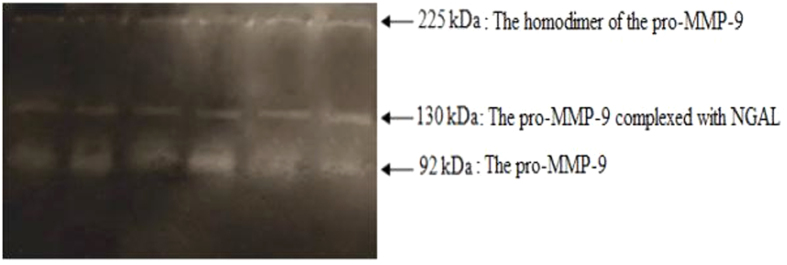
Representative gelatin zymograms of serum samples with MMP-9 forms.

A representative gelatin zymogram of serum samples with all forms of MMP-9 commonly found in human is shown in Fig. 4. Three forms of MMP-9 were detected, including the homodimer of the pro-MMP-9 form (225 kDa), the pro-MMP-9 complexed with neutrophil gelatinase-associated lipocalin (NGAL) form (130 kDa) and the monomeric form of the pro-MMP-9 (92 kDa). The results of densitometry analysis are indicated in Supplementary Figs S2–S4 respectively. In our data, a statistically significant increase in protein activity of homodimer pro-MMP-9 (225 kDa) was noted in patients (25.7 ± 2.92%) compared with healthy subjects (16.78 ± 3.15%). The serum activity of the pro-MMP-9 complexed with NGAL (130 kDa) was significantly higher in patients with PU (9.37 ± 3.17%) compared to controls (6.2 ± 1.32%). Furthermore, a statistically significant increase in mean serum activity of the monomeric of pro-MMP-9 form (92 kDa) was present in patients with PU (58.32 ± 16.99%) than in controls (34.71 ± 10.33%).

### Allele and genotype frequencies of the MMP9 -1562C/T polymorphism and risk of PU

In the current study, evaluation of Hardy-Weinberg equilibrium showed that the genotype frequencies of the MMP9 -1562C/T polymorphism were in Hardy-Weinberg equilibrium in both groups (p ≤ 0.05) (Supplementary Table S2).

**Figure 5:**
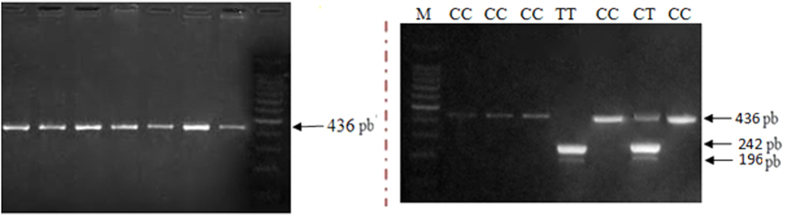
MMP9-1562C/T (rs3918242) genotyping by PCR-restriction fragment length polymorphism.

Three distinct genotypes, based on the two alleles found, CC, CT and TT were observed in all analyzed populations (Fig. 5). The frequency distributions of different MMP9-1562C/T genotypes and alleles are summarized in Table 3.

Comparison of allele T frequencies showed statistically significant difference between patients suffering from PU in comparison with healthy subjects (odds ratio (OR) = 0.55, 95% confidence interval (CI) = [0.33–0.92], p = 0.021). The combined risk genotype (CT + TT) frequencies of SNP -1562 MMP9 also differed significantly between patients and controls (OR = 0.53, 95% CI = [0.3–0.93], p = 0.025). Therefore, the results indicated that the MMP9-1562C/T polymorphism is associated with PU, and that the minor T allele may be a protective factor for this condition.

## Discussion

PU causes serious complication that lead to severe or intolerable pain and increased morbidity and mortality rates affecting approximately 3 million adults^25^,^26^. These elevated rates indicate the existence of a problem that merits investigation, since it is known that in the majority of cases PU can be avoided by identification of risk factors and initiation of preventative measures^27^. Furthermore, PU is wound with multifactorial origins in which the cellular and molecular mechanisms contributing to massive destruction of deeper tissues need to be more defined. The present study was based on the assumption to evaluate the possible role of the predisposing factors in PU.

Although there is little reference in the literature regarding a relationship between PU and gender, among 15 studies included in the systematic review of Coleman *et al*. only four of them have shown an association between PU and gender^28^. In more recent studies, Ek *et al*. observed a greater occurrence of PU in women but gender is not considered to be a risk factor^29^ whereas Brandeis *et al*. find male gender was a risk factor for conversion and complications of PU^30^.

In 2004, Blanes *et al*. analyzed the relationship between age, gender and PU risk factors in a group of hospitalized patients with PU, they found a mean age of 64 years or women were 67.9 years old and were slightly older than the men 60.4 years^31^. In fact, older people are more susceptible to developing skin wounds because of age related skin changes, immobility that increases the risk of incontinence, cognitive impairment, lose muscle mass and gain weight.

Interestingly, our patients group comprises 44% of subjects over 60 years of age, 7% of them are aged more than 80 years and we state that men (74%) are at a higher risk of developing PU than women.

In addition, bedridden patients and those with cognitive impairment are more susceptible to PU development^32^. Indeed, almost all chronic wounds occur in a host with a predisposing condition such as venous disease, arterial and vascular compromise and neuropathy disease^33^. In the present study, neuropathy and cardiovascular diseases (cerebral vascular accident, heart failure, diabetes, hypertension and renal insufficiency) were the most frequently seen pre-existing diseases in the patient group.

Although the prevention of PU is a multidisciplinary responsibility, nutrition plays a fundamental part in wound healing, with malnutrition, dehydration and recent weight loss identified as independent risk factors for the development of PU. While the low levels of cholesterol, hemoglobin, albumin and micronutrients (vitamins A and C and zinc) are often quoted as independent predictors of PU risk^28^.

In particular, albuminemia remains an unreliable indicator of nutritional status because it may be more related to inflammation or hydration status than to malnutrition^34^. Other studies showed a lower albumin to be related to higher stages of PU. A low serum albumin has been suggested to promote PU formation through interstitial edema, reduced tissue oxygenation and nutrient flow^35^. In fact, our data showed that 41% of patients with hypoalbuminemia.

Subsequently, 48% of our patients with PU were found to be at severe nutritional risk according to NRI score. However, this is in agreement with the higher proportion of patient (52%) were found to be at severe nutritional condition according to PINI score (21 < PINI < 30 and PINI > 30). Our findings are in agreement with the results of previous studies displaying higher risk of various complications in malnourished patients with PU as compared to normal volunteers non-malnourished^36^,^37^. Poor nutritional status can lead to delay wound healing and increase risk of infection.

Chronic wounds are colonized by polymicrobial flora, originating from the external environment, local skin flora, the enteric tract, the vagina and oral mucosa^38^. In our study, we have isolated 10 bacterial strains, proteus mirabilis was the most common germ. Microflora of chronic wound fluctuates over time. Thus, on young wound predominantly gram-positive cocci. After a few weeks, the bacilli are gram negative which colonizes the wound.

The proliferation of gram-negative bacilli often manifests as a color change of wound, and they appear to play a particularly important role during the detersion phase. However, infections by gram-positive cocci respect the appearance of the wound. They evolved towards the formation of a purulent pocket. This pocket can cause a fever and also be the starting point for the bacterial invasion of adjacent tissues^38^.

Indeed, infection is a common complication of PU and may be local (cellulitis, joints and bones) or distant. Joint infections (septic or infectious arthritis) can damage cartilage and tissue within days, whereas bone infections (osteomyelitis) may reduce the function of joints and limbs for years if not treated. One of the greatest dangers of an advanced PU, sepsis occurs when bacteria from a massive infection enter the bloodstream through broken skin and spread throughout the body. Such infections can lead to life-threatening complications that can cause shock and organ failure^39^. In the present study and according to their medical records, 21 patients have sepsis and clinical signs that the PU may be infected include malodorous, pain, purulent exudates and excessive bleeding in the ulcer.

Moreover, chronic wounds lead to persistent bacterial populations that are typically arranged into highly organized biofilms. The importance of biofilms in chronic wounds has been recently reviewed in detail^40^. High microbial burden leads to the presence of toxins or other factors within and around the wound. This process can lead to an excessive inflammatory reaction involved in the development of PU and other subsequent complications.

The inflammatory response can ultimately lead to the development of complex lesions and encourages a sustained release of cytotoxic enzymes, pro-inflammatory cytokines (interleukin-1, tumour necrosis factor-alpha) and free oxygen radicals^41^. This was illustrated by the significantly higher serum levels of nontraditional markers of subclinical inflammation, including CRP and α1‐acid glycoprotein, in our group of patients compared to controls. Additionally, inflammation is a key component against infection upon tissue injury in the pathogenesis of PU and is thought to play a key promoting role in oxidative stress^42^.

Oxidative stress is a state of altered physiological equilibrium within a cell or tissue/organ, defined as a condition arising when there is a serious imbalance between the levels of free radicals in a cell and its antioxidant defenses in favor of the former^43^. It not only causes hazardous events such as lipid peroxidation and oxidative deoxyribonucleic acid (DNA), ribonucleic acid (RNA) and proteins damage, but also physiologic adaptation phenomena and regulation of intracellular signal transduction^44^,^45^.

In our data, investigation about oxidative stress parameters shows a decreased serum TBARS level, as a lipid peroxidation marker, and a drop in the TAS in patients with PU compared to healthy subjects. According to our observation, Taylor *et al*. have considered that the TBARS levels measured were predictive of the development of PU independently of lipids levels (total cholesterol, HDL-C, LDL-C, and triglycerides), blood pressure, inflammatory markers, age and body mass index (BMI)^46^.

Furthermore, our patients with PU have significantly hyperhomocysteinaemia. Homocysteine reduces the transition metal ion (Mn+) to generate a thioyl radical (Hcy°). It is thought to react with homocysteine to generate a free radical intermediate that reduces oxygen to superoxide anion and then to peroxide hydrogen generation. The imbalance between pro-oxidant and antioxidant systems can be aggravated by homocysteine which is readily oxidized^47^.

Interestingly, several studies have investigated the expression and activity of MMP-9 in many physiological and pathological processes^15^,^18^,^20^. When present in the bed skin in large quantities, and for long periods in the wrong places, it begins to degrade the extracellular matrix, inhibit cell migration and prevent wound closure^48^. However, Gumieiro *et al*. reported that pro-MMP-9 was associated with gait status recovery 6 months after hip fracture but was not associated with PU and mortality in hip fracture patients^49^. Some studies analyzing fluids and wound biopsies collected from PU patients showed overexpression of the active forms MMP-9. These data suggest that this protease could destroy the growth factors, receptors and extracellular proteins essential for the healing of PU^19^,^21^,^50^.

In the present study, our data revealed that the serum activity of the pro-MMP-9 increased more in the patients suffering from PU than the healthy subjects. This phenomenon can be explained by certain cells that secrete an excessive amount of MMP-9 into the circulation and also the decreased transformation from the inactive form to the active form of MMP-9. To our knowledge, our findings gave unprecedented evidence that the overexpression of MMP-9 protein is associated with PU. As a result, the serum pro-MMP-9 is a predictor of the wound healing process.

Because the expression of MMP-9 is primarily controlled by the rate of gene transcription, differences in promoter activity should be reflected in levels of MMP-9. Therefore, it is important to understand the factors that control the transcriptional response of this gene. Several SNP have been identified in the MMP-9 gene, including polymorphisms in the proximal promoter^51^.

Among these polymorphisms, the -1562C/T polymorphism in the MMP-9 promoter may reduce the rate of transcription by inhibiting protein binding, which down regulates MMP-9 expression^52^,^53^. Blankenberg *et al*. showed that -1562C>T polymorphism has an influence on MMP-9 transcription and that this effect is translated into differences in MMP-9 protein level and activity between individuals of different genotypes^54^.

A positive association of SNP -1562C>T in the MMP-9 gene has been reported with many diseases^53^. However no previous study investigated the relationship between PU and MMP-9 polymorphisms. In the present study, we assessed the association between MMP9 -1562C/T polymorphism and PU in Tunisian patients. Interestingly, the distribution of MMP9 -1562C/T genotypes and alleles frequencies were significantly different between PU patients and controls with the T allele conferring a lower risk for PU. These results suggest that a common functional MMP-9 promoter polymorphism (rs3918242) protects against PU and indicate that a rather modest change due to an SNP may result in considerable biological differences.

In summary, in medicine we must know the causes of disease and health. PU requires a large variety of medical care and patient with PU in Tunisia hospitals suffer from lack of healthcare and consciousness. The present data suggest that PU is a complex clinical problem with multiple influencing factors. Furthermore, the conventional risk factors such as age, gender, serum lipid levels and oxidative stress parameters showed different in our patient and control groups. Various molecules found in serum may be reliable markers for early diagnosis of PU. Measurements of albumin, CRP and homocysteine have been proposed as biochemical parameters able to increase diagnostic certainty.

In addition, MMP-9 is a gelatinase that exerts a variety of immunoregulatory activities and is thought to play a critical role in a number of pathophysiological conditions like PU and its acute complications. Therefore, an excess in MMP-9 appears to be an accurate prognostic markers of wound repair. Moreover, our study provided potential evidence of the association between MMP9-1562C/T polymorphism and the risk of PU, and supported the hypothesis that the MMP9-1562 T allele variant might be a genetic marker for the risk of this disease. So the missing determination of plasma levels of MMP-9 and testing only SNP in the investigated gene represent the limitations of this study.

Of interest, these variables can be used as references to determine the priority needed to be given to some preventive measures and treatments and to identify the patients at risk. Future research should better establish the signaling pathway as well as predisposing factors to systemic infection and other studies need to be performed to investigate thoroughly the underlying cellular mechanisms and whether this knowledge can be used to develop novel therapeutic approaches.

## Methods

### Study population

A total of 313 individuals, comprising 100 patients suffering from PU and 213 healthy controls, were enrolled in this study. Controls and patients were selected from the same population living in the central coast of Tunisia and including only unrelated subjects. 100 patients suffering from PU that met the following inclusion criteria: presenting at least a wound and confirmed diagnosis of PU, aged ≥18 years old, bedridden, not feeds only and without trophic and mental disorders. Exclusion criteria: pediatric study population, age >90 years old, allergy to wound products, malignant origin, obstetric and psychiatric patients were excluded^55^. These patients were recruited from the medical care units (emergency, orthopedic, physical medicine and surgical) at three University Regional hospitals in Tunisia (Farhat Hached/Sahloul Sousse, and Fattouma Bourguiba Monastir). Healthy individuals (n = 213) were recruited from the outpatient services at the Farhat Hached Hospital (Sousse, Tunisia) and they were considered clinically free of PU and tissue necrosis. Informed consent was obtained from each participant or his legal representative. The study was approved by the national committee for medical and research ethics of the university hospital Farhat Hached in sousse - Tunisia and complied with the ethical principles of the WMA Declaration of Helsinki. The methods carried out in the study are in accordance with the approved relevant guidelines and regulations.

### Blood collection and biochemical methods

Fasting venous blood samples were collected and immediately centrifuged at room temperature for 10 minute (mn) at 3000 rpm. Plasma levels glucose, total serum cholesterol, serum triglycerides, HDL-C and a renal profile (urea, creatinine and uric acid) levels were measured by a standard spectrophotometric method using reagents in a fully automated analyzer (Randox Antrim, UK; CX5 and CX9-BECKMANN). LDL-C was determined by Friedewald formula. Positive acute phase proteins such as CRP and α1‐acid glycoprotein were measured using immunoturbidimetric method (COBAS INTEGRA 400 Roche). Markers of nutritional status, albumin and prealbumin (negative acute phase proteins) were measured using the dry chemistry method (BN prospec, siemens). Total homocysteine in serum was measured by the Abbott homocysteine assay (AXSYM).

Evidence of lipid peroxidation was detected by measurement of TBARS in serum according to the fluorimetric method of Yagi^56^. The principle for the TBARS is based on TBA reacting with the LDL oxidation product, malondialdehyde (MDA), to produce TBA-reactive substances. Serum TBARS levels were measured at 515 nm excitation and 553 nm emission with the reference to a standard MDA solution prepared by acid hydrolysis of 1,1,3,3-tetraethoxypropane. The TAS was measured by colorimetric method at 600 nm according to the method of Miller *et al*. (Cat. No. NZ 2332; Randox Labs Ltd., Crumlin, UK). This test can evaluate the total capacity of all antioxidants found in biological sample to neutralize the oxidative action of free radicals^57^.

### Swab sample collection

Only one wound per patient was cultured by swab to determine the bacterial species of the infection and help guide antibiotic therapy. The representative sample is collected before topical or systemic antibiotics are initiated and pain assessment should be conducted prior to wound procedures such as dressing changes and debridement. Immediately, the quantitative results of surface swab culture were evaluated at the microbiology laboratory of Farhat Hached Hospital in Sousse.

### Nutritional status assessment

Malnutrition was determined by the presence of at least one of the following elements based on the previous studies in hospitals. Anthropometric evaluation was performed by the usual parameters: body weight (BW, Kg), body height (BH, m), and BMI was calculated using the formula: BMI (Kg/m^2^) = BW (Kg)/BH^2^ (m^2^). Biochemical evaluation of negative acute phase protein such as albumin (chronic marker of nutritional status) was measured by immunoenzymatic method.

Patients were scored according to Nutritional Risk Index (NRI) [NRI = (1.489× albumin) + (41.7× present weight/ideal body weight)] and categorized as follows: severe risk (NRI < 83.5), moderate risk (83.5 < NRI < 97.5) and no risk (NRI > 97.5). Prognostic Inflammatory and Nutritional Index (PINI) is a simple clinical assessment [PINI = α1‐acid glycoprotein × CRP/albumine × prealbumin] and classificated as follows to 4 categories: normal (1 < PINI score < 10), mild malnutrition (11 < PINI score < 20), severe malnutrition (21 < PINI score < 30) and risk for death when PINI score >30. These scores gained in popularity as it uses an objective rather than subjective measurements to determine nutritional risk in hospitalized patient populations^58^,^59^.

### Mortality of patients with PU

For information on post discharge mortality, the hospital database was interrogated to determine patient’s vital status. Individuals who were noted to be alive on the database were contacted at least after 3 months after discharge via telephone to verify their status.

### Gelatin Zymography of MMP-9

Previously, the MMP-9 proteolytic activity was measured by a relatively easy yet powerful technique known as zymography^49^. Each serum sample is mixed with 2x sample buffer consisting of 0.5 M Tris, pH 6.8, 100% glycerol and 0.5% bromophenol blue. Electrophoresis was carried into the wells of 8% sodium dodecyl sulfate-polyacrylamide gel (SDS-PAGE) containing 1% gelatin out using a mini protean II system (Bio-Rad) at 90 V for 2 hours. The run is complete when the bromophenol blue tracking dye reaches the bottom of the gel. After electrophoresis, the gel washed two steps in renaturing solution through 2.5% Triton X-100 and then with 50 mM Tris pH 8.4 on a rotatory shaker. For maximum sensitivity, each gel was incubated overnight (37 °C) in a developing solution and protect them with plastic film. After the water bath, the gel should be stained 2 times with 0.1% coomassie brilliant blue and then be stained twice more with bleach solution (40% methanol and 7% acetic acid) until reaching maximum objection to detect clear bands against a blue background of undegraded substrate. Moreover, the stained gels were scanned on Canon-Cano Scan LIDE 25 F91011 and the density of stained gelatinolytic band was measured by ImageJ or that rate was expressed as a percentage.

### Genotyping of single nucleotide polymorphism (-1562C>T) MMP9

Genomic DNA was isolated from peripheral white blood cells using standard salting-out procedures. Genotyping of MMP9-1562C/T (rs 3918242) polymorphism in the study population (313 individuals) was performed using a polymerase chain reaction and restriction fragment length polymorphism (PCR-RFLP) method according to Wang *et al*.^60^ with the following changes: a 18 μl reaction volume mixture was prepared using sterile water, 3.6 μl of 5X buffer (50 mM KCl, 20 mM Tris-HCl, pH 8.4), 0.72 μl of 25 mM MgCl_2_, 0.36 μl of 10 uM of each allele specific primers [sense primer (5′-GCCTGGCACATAGTAGGCCC-3′) and antisense primer (5′-CTTCCTAGCCAGCCGGCATC 3′)], 0.36 μl of 20  mM dNTP, GoTaq^®^ Flexi DNA Polymerase (0.09 U) (Promega, Madison, WI, USA), and 1 μl of 100 ng of genomic DNA.

Briefly, the PCR-RFLP consisted of a 5 mn denaturation step at 94 °C followed by 35 cycles at 94 °C for 30 second (s), 63.5° for 30 s, 72 °C for 50 s, and a final extension at 72 °C for 10 mn.

The PCR products were digested overnight with 2 units of SphI (Jena Bioscience GmbH, Germany) at 37 °C and were analyzed by electrophoresis on a 2% agarose gel then stained with ethidium bromide before genotype assessment under ultraviolet light. The fragment sizes were 436 base pair (bp) (undigested) for the C allele whereas 194 and 242 bp for the polymorphic variant (T allele).

### Data analysis

Database management and statistical analyses were carried out using Statistical Package for the Sociological Sciences (SPSS) version 18.0 and Epi info 6.0 softwares. Results are presented as means ± SD or the median and percentages. Means were compared using Student test. The significance threshold was set at 5% (p < 0.05).

## Additional Information

**How to cite this article**: Latifa, K. *et al*. Evaluation of physiological risk factors, oxidant-antioxidant imbalance, proteolytic and genetic variations of matrix metalloproteinase-9 in patients with pressure ulcer. *Sci. Rep.* **6**, 29371; doi: 10.1038/srep29371 (2016).

## Supplementary Material

Supplementary Information

## Figures and Tables

**Table 1:** Assessment of degree of malnutrition according to analysis of serum albumin level, BMI and indexes in patients suffering from PU.

Parameters	Albumin (g/l) (27.91 ± 8.15)	BMI (Kg/m^2^) (20.92 ± 3.29)	NRI (71.83 ± 29.11)	PINI (139.63 ± 342.31; M = 27.36)
Degree				
Normal	30 < Alb < 35 36.51 ± 4.74 34%	25 < BMI ≤ 30 32.35 ± 1.31 6%	NRI > 97.5 106.26 ± 7.55 29%	1 < PINI < 10 4.87 ± 2.97 26%
Moderate	25 < Alb < 30 28.38 ± 1.59 25%	18.5 < BMI < 25 21.23 ± 1.45 54% (mildly impaired)	83.5 < NRI < 97.5 90.83 ± 4.01 23%	11 < PINI < 20 14.87 ± 3.27 22%
Severe	Alb < 25 20.35 ± 4.66 41%	16.5 < BMI < 18.5 17.98 ± 0.41 33% (Moderately)	NRI < 83.5 52.73 ± 20 48%	21 < PINI < 30 25.8 ± 3.3 13% (Severely)
		BMI < 16.5 16.38 ± 0.6 7% (Severely)		PINI > 30 281.82 ± 459.73 (M = 89.4) 39% (Risk for death)

Abbreviations: Albumin (Alb), Body mass index (BMI), Median (M), Nutritional risk index (NRI), Prognostic inflammatory and nutritional index (PINI).

**Table 2:** The clinical and laboratory variables of the participants.

Variables	Patients (n = 100)	Controls (n = 213)	P
Women/Men	26/74	88/125	0.001
Age, years	55.5 ± 20	51.5 ± 17	0.016
BMI, kg/m^2^	20.92 ± 3.29	24.09 ± 3.36	0.000
Glucose, mmol/l	7.04 ± 3.44	6.03 ± 2.12	0.004
Urea, mmol/l	6.95 ± 6.13	4.68 ± 1.79	0.000
Creatinine, μmol/l	117.86 ± 162.97 (M = 66.5[α_1_ 48.5; α_2_ 96.5])	74.98 ± 81.84 (M = 62 [α_1_ 54; α_2_ 75])	0.007
Uric acid, μmol/l	202.68 ± 143.68	214.01 ± 75.8	0.41
Protide, g/l	64.33 ± 10.86	74.37 ± 6.17	0.000
Cholesterol, mmol/l	3.25 ± 1.2	4.67 ± 1.03	0.000
Triglyceride, mmol/l	1.54 ± 0.72	1.33 ± 0.81	0.048
HDL-C, mmol/l	0.76 ± 0.46	2.01 ± 1.29	0.000
LDL-C, mmol/l	1.38 ± 1.15	2.23 ± 1.04	0.000
CRP, mg/l	98.69 ± 76.93	6.31 ± 6.25	0.000
α1‐acid glycoprotein, g/l	1.85 ± 0.62	1 ± 0.5	0.000
Albumin, g/l	27.91 ± 8.15	40.79 ± 8.43	0.000
Prealbumin, g/l	0.13 ± 0.11	0.28 ± 0.15	0.000
Homocysteine, μmol/l	27.22 ± 12.50	10.46 ± 3.64	0.003
TBARS, μmol/l	0.47 ± 0.31	1.11 ± 1	0.000
TAS, mmol/l	1.48 ± 0.72	1.76 ± 0.21	0.000

Abbreviations: Body mass index (BMI), C-reactive protein (CRP), High density lipoprotein cholesterol (HDL-C), Low density lipoprotein cholesterol (LDL-C), Median (M), Thiobarbituric acid reactive substances (TBARS), Total antioxidant status (TAS).

**Table 3:** Distribution of MMP9-1562C/T alleles and genotypes in patients with PU and control groups.

	Patients, n (%) (n = 100)	Controls, n (%) (n = 213)	P-value	OR	95% CI
Allele T	21 (10.5%)	75 (17.61%)	**0.021**	0.55	[0.33–0.92]
CC	79 (79%)	142 (67%)		1	
CT	21 (21%)	67 (31.44%)	**0.05**	0.58	[0.33–1.02]
TT	0	4 (1.9%)		–	–
CT + TT	21 (21%)	71 (33%)	0.025	0.53	[0.3–0.93]

Abbreviations: Confidence interval (CI), Odds ratio (OR).
