# Supplementary material for: Evaluation of physiological risk factors, oxidant-antioxidant imbalance, proteolytic and genetic variations of matrix metalloproteinase-9 in patients with pressure ulcer
Source: Sci Rep. 2016 Jul 11;6:29371. doi: 10.1038/srep29371 (PMC4942564; doi:10.1038/srep29371)
Supplement: Supplementary Information [file srep29371-s1.pdf]

**Supplementary**

Title: **Evaluation of physiological risk factors, oxidant–antioxidant imbalance, proteolytic and genetic variations of matrix metalloproteinase-9 in patients with pressure ulcer**

Khlifi Latifa[1*], Sahli Sondess[1], Graiet Hajer[1], Ben-Hadj-Mohamed Manel [1], Khelil Souhir[1], Bouzidi Nadia[1], Jaballah Abir[1], Ferchichi Salima[1] and Miled Abdelhedi [1].

[1] Biochemistry Laboratory CHU Farhat HACHED, Sousse, Tunisia

__Supplementary Tables__

## Supplementary

**Supplementary Table S1:** Subject demographics and pressure injury risk assessment

| | |
|---|---|
| **The enrolment / N° of patient** | |
| Hospital Sahloul Sousse ( Ward) | **75** |
| - Emergency | 35 |
| - physical medicine | 18 |
| - Orthopedic | 16 |
| - Surgical | 6 |
| Hospital Fattouma Bourguiba Monastir | **16** |
| - Emergency | 8 |
| - Orthopedic | 5 |
| - Surgical | 3 |
| Hospital Farhat Hached Sousse | **9** |
| - Emergency | 7 |
| - Surgical | 2 |
| **The occurrence / N° of patient** | |
| - Home | 38 |
| - During hospitalization in department | 46 |
| - During hospitalization in another department | 13 |
| - Other | 3 |
| **Health conditions / N° of patient** | |
| - Paralysis | 41 |
| - Cardiovascular disease | 38 |
| - Coma | 27 |
| - Polytrauma | 24 |
| - Dementia/alzheimer's | 4 |
| **PU risk assessment / N° of patient** | |
| - Braden low risk | 6 |
| - Braden moderate risk | 30 |
| - Braden high risk | 41 |
| - Risk very high risk | 23 |
| **Medications** | |
| Tienam $_{500\ mg}$ - Amiklin $_{500\ mg}$ - Tazo $_{200mg}$ - Rifampicine $_{600\ mg}$ - Claforan $_{1g}$ - Augmentin - Silvaderm. Clean the wound by: Saline solution - Betadine - Sofra tulle | |
| **Locations of pressure ulcer / N° of patient** | |
| - Sacrum | 42 |
| - Trochanter | 27 |
| - Ischium | 26 |
| - Heels | 51 |
| - Back of head | 14 |
| - Shoulder | 13 |
| - Tendon | 5 |
| - Headset | 4 |
| - Toenail | 1 |
| **Stages of pressure ulcer / N° of patient** | |
| - Stage 1 | 8 |
| - Stage 2 | 17 |
| - Stage 3 | 27 |
| - Stage 4 | 48 |
| **Mortality** | **31** |

**Supplementary**

**Supplementary Table S2:** Hardy-Weinberg equilibrium test for the MMP9-1562 C/T polymorphism in patients and control groups

| | Patients group | | Controls group | |
|---|---|---|---|---|
| Genotypes | Observed value | Expected value | Observed value | Expecte value |
| CC | 79 (79%) | 80.1 (80.1%) | 142 (66.66%) | 144.6 (67.89%) |
| CT | 21 (21%) | 18.8 (18.8%) | 67 (31.44%) | 61.8 (29.01%) |
| TT | 0 | 1.1 (1.1%) | 4 (1.9%) | 6.6 (3.1%) |
| | PU group: $\chi2$ =1.372, P-value =0.2407; Controls group: $\chi2$ =1.508, P-value =0.219 | | | |

## Supplementary Figures

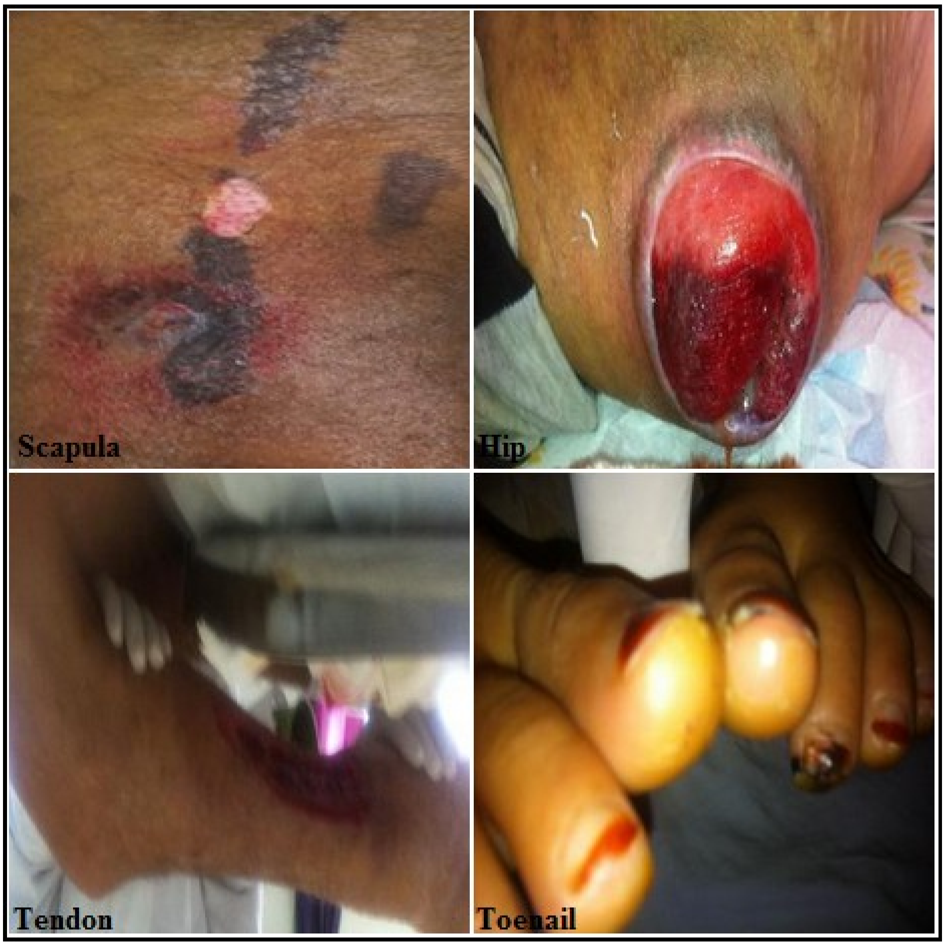


**Supplementary Fig. S 1:** Other locations of pressure ulcer

**Supplementary**

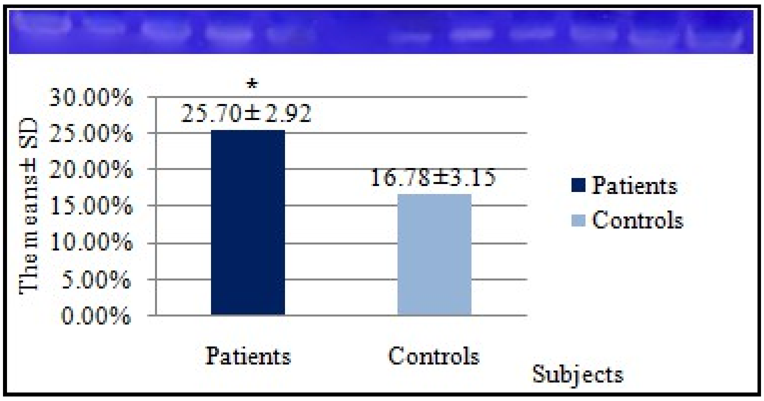


**Supplementary Fig. S 2**: Serum activity of the homodimer pro-MMP-9 (225 kDa)

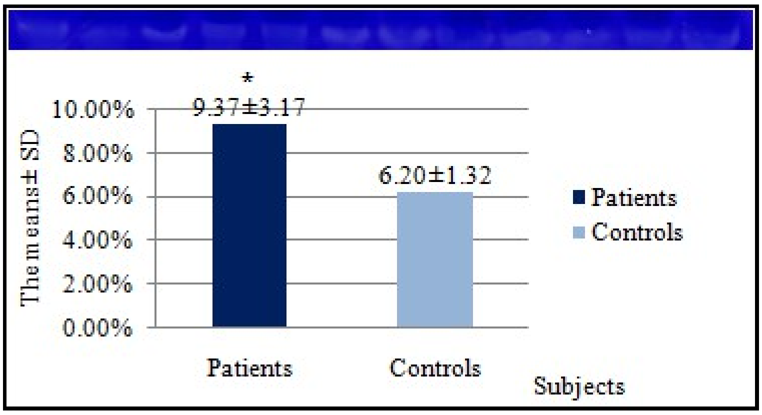


**Supplementary Fig. S 3**: Serum activity of the pro-MMP-9 complexed with NAGL (130 kDa)

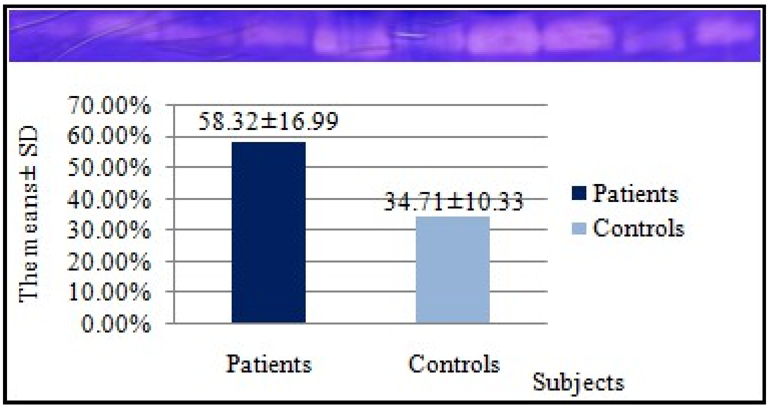


**Supplementary Fig. S 4**: Serum activity of the monomeric form of pro-MMP-9 (92 kDa)
